# The Impact of a Swimming Training Season on Anthropometrics, Maturation, and Kinematics in 12-Year-Old and Under Age-Group Swimmers: A Network Analysis

**DOI:** 10.3389/fspor.2022.799690

**Published:** 2022-02-21

**Authors:** Júlia Mello Fiori, Paulo Felipe Ribeiro Bandeira, Rodrigo Zacca, Flávio Antônio de Souza Castro

**Affiliations:** ^1^School of Physical Education, Physiotherapy and Dance, Aquatic Sports Research Group (GPEA), Universidade Federal do Rio Grande do Sul, Porto Alegre, Brazil; ^2^Department of Physical Education, Universidade Regional do Cariri–URCA, Crato, Brazil; ^3^Aftergraduate Program in Physical Education - Universidade Federal do Vale do São Francisco–UNIVASF, Petrolina, Brazil; ^4^Research Center in Physical Activity, Health and Leisure (CIAFEL), Faculty of Sports, University of Porto (FADEUP), Porto, Portugal; ^5^Laboratory for Integrative and Translational Research in Population Health (ITR), Porto, Portugal

**Keywords:** analysis, digital technology, long-term athletic development, biomechanics, technique, anthropometrics, exercise physiology, maturation

## Abstract

Understanding fluctuations and associations between swimming performance-related variables provide strategic insights into a swimmer's preparation program. Through network analysis, we verified the relationships between anthropometrics, maturation, and kinematics changes (Δ) in 25-m breaststroke (BREAST) and butterfly (FLY) swimming performance, before and after a 47-week swimming training season. Twenty age-group swimmers (*n* =11 girls: 10.0 ± 1.3 years and *n* = 9 boys: 10.5 ± 0.9 years) performed a 25-m all-out swim test (T25) in BREAST and FLY techniques, before and after 47 weeks. Three measures of centrality, transformed into a z-score, were generated: betweenness, closeness, and strength. Data were compared (*t*-test) and effect sizes were identified with Hedges' g. Large effect sizes were observed for swimming performance improvements in BREAST (32.0 ± 7.5 to 24.5 ± 3.8 s; g = 1.26; Δ = −21.9 %) and FLY (30.3 ± 7.0 to 21.8 ± 3.6 s; g = 1.52; Δ = −26.5 %). Small to moderate effect sizes were observed for anthropometric changes. Moderate effect size was observed for maturity offset changes (−2.0 ± 0.9 to −1.3 ± 1.0; g = 0.73; Δ = 50.9 ± 281 %). Changes in maturity offset, stroke rate (SR), and stroke length for both BREAST and FLY swimming speeds were highlighted by the weight matrix. For betweenness, closeness, and strength, changes in arm span (AS) (BREAST) and stroke length (FLY) were remarkable. The dynamic process of athletic development and the perception of complexity of fluctuations and associations between performance-related variables were underpinned, particularly for simultaneous swimming techniques in age-group swimmers.

## Introduction

The performance of age-group swimmers improves based on the relationships among technical, physical and anthropometric factors, which are characterized by a complex adaptive system (CAS). Whereas there is body growth, drag and propulsion change, i.e., the swimming performance related factors may be deeply influenced by the anthropometric characteristics. Not conceptualizing swimming performance as a CAS phenomenon is a limitation that should be avoided (Morais et al., 2014; Ferreira et al., 2019; Zacca et al., 2020b). To complement the traditional statistical approaches, a multivariate model (as global as possible) could bring new insights on changes in swimming performance, particularly during a training season. Network analyses can provide a global view of this multivariate phenomenon, that is, accessing both linear and nonlinear relationships between swimming performance-related variables (Holland, 2006; Schmittmann et al., 2013; Goethel et al., 2020; Guido, 2020; Pol et al., 2020).

There is a scientific and practical interest in individual maturation and the ideal period to start working on individual physical skills in long-term athletic development (LTAD; Lätt et al., 2009; Dias et al., 2012; Collins et al., 2019). Longitudinal studies can provide relevant insights (Mitchell et al., 2020; Zacca et al., 2020a), but there are few longitudinal studies based on 12-year-old and under age-group swimmers (Morais et al., 2014, 2020; Ferreira et al., 2019). It is well reported that anthropometrics and maturation can affect athletic development in age-group swimmers (Dias et al., 2012; Moreira et al., 2014; Morais et al., 2020), with enhanced swimming performance being observed even after detraining periods (Meylan et al., 2014; Moreira et al., 2014). The interplay between maturation and training response should be considered by coaches (Muller et al., 2017; Pichardo et al., 2018), but most previous analyses are fragmented instead of considering the interdependence among the selected variables (Goethel et al., 2020). Monitoring maturity status through age at peak height velocity (PHV) can be an effective, practical, and noninvasive approach (Beunen and Malina, 1988; Mirwald et al., 2002; Philippaerts et al., 2006; Malina, 2016).

The human body consists of several interdependent systems, and multiple factors can affect the ability to swim fast. Identifying which factors are important for fast swimming and how to maximize these factors for performance improvements requires understanding the existing network relationships. Interventions and/or phenomena in a specific system can trigger responses in another apparently unrelated system (Goethel et al., 2020). By applying network analysis, it is possible to identify the effects and interactions of each variable in a global approach, especially when considering the effects of changes in variables over time and their possible effects on changes in other variables. We therefore performed a global analysis using the changes in representative variables to assess which variables, in relation to their changes, could be more important for swimming performance. So, the aim of this study is to identify the relationships between changes in anthropometrics, maturation, and swimming kinematics on breaststroke (BREAST) and butterfly (FLY), before and after a 47-week swimming training season in 12-year-old and under age-group swimmers.

## Methods

### Participants

Twenty age-group swimmers participated in this study. Age for girls (*n* = 11) and boys (*n* = 9) were, respectively, 10.0 ± 1.3 (4.0) and 10.5 ± 0.9 (2.5) years (mean ± SD, and range), respectively. The participants were engaged in swimming training for at least 12 months, swimming 3 to 5 times per week, 1.000 to 2.000 m per session and had been engaged in a swimming training program for at least for 6 months. During the 47 weeks, the best performance in the 50-m front crawl was, for girls and boys, respectively, 40.2 ± 5.4 (min–max: 36.6–43.8) and 36.8 ± 6.5 (31.8–41.9) s.

### Procedures

All swimmers were evaluated during two identical testing sessions: (i) before the training season, that is, during the first week of training after the summer vacation; and (ii) after 47 weeks, at the end of the last macrocycle of the season. First, the anthropometric profile was obtained, which was consisted by height (HE), arm span (AS), total body mass (BM), and sitting height (SH). After an approximately 400-m moderate-intensity warmup, swimmers performed two 25-m all-out swim tests (T25; randomized order), one in breaststroke (BREAST), and one in butterfly (FLY), whereas kinematic variables were collected manually by one trained and experienced evaluator (Hay and Guimarães, 1983) using a stopwatch (CASIO HS-70w, Japan). We used manual data collection to assess technical variables (kinematic) since it is feasible for swimming coaches in their daily training. The performance of 25 m (s), time (s) to swim intermediate 10 m, and time (s) to perform three consecutive stroke cycles along the intermediate 10 m were collected manually (Hay and Guimarães, 1983), with kinematic variables being calculated according to Equations 1–4:


(1) $$\begin{array}{r} \;\text{Swimming speed }\left(\text{m}.{\text{s}}^{-1}\right):\text{v }=\;10\;\text{m}.\text{time of }10\;{\text{m}}^{-1} \end{array}$$

(2) $$\begin{array}{l} \text{ Stroke rate }(\text{cycles}.{\text{s}}^{-1}):\text{SR }=\;3\text{ stroke }\\ \text{ cycles}.\text{time of 3 stroke }{\text{cycles}}^{-1} \end{array}$$

(3) $$\begin{array}{r} \;\text{Stroke length (m): SL = v}.{\text{SR}}^{-1} \end{array}$$

(4) $$\text{Stroke index}({\text{m}}^{2}.{\text{s}}^{-1}):\text{SI = v}.\text{SL}$$

Then, stroke rate (SR) was multiplied by 60 to obtain SR in cycles·min^−1^.

#### Anthropometrics

Height, AS, BM, and SH were measured (Heyward and Stolarczyk, 1996), and leg length (LL) was estimated as stature minus sitting height (Mirwald et al., 2002). For BM, a weighting scale (TECHLINE^®^, Brazil) was used. For HE, AS, SH, and LL, a 250-cm tape (VONDER^®^, Brazil) was used.

#### Maturation

Maturity offset equations (Mirwald et al., 2002) were applied with anthropometrics and age data. The equations for boys and girls are, respectively (Equations 5 and 6):


(5) $$\begin{array}{r} BMO=-9.236+\left[0.0002708^{*}\left(LL^{*}SH\right)\right]-\left[0.001663^{*}\left(A^{*}LL\right)\right]\\ \;+\left[0.007216^{*}\left(A^{*}SH\right)\right]+\left\{0.02292^{*}\left[{\left(\frac{\text{BM}}{\text{height}}\right)}^{*}100\right]\right\} \end{array}$$

(6) $$\begin{array}{r} GMO=-9.376+\left[0.0001882^{*}\left(LL^{*}SH\right)\right]+\left[0.0022^{*}\left(A^{*}LL\right)\right]\\ \;+\left[0.005847^{*}\left(A^{*}SH\right)\right]-\left[0.002658^{*}\left(A^{*}BM\right)\right]\\ \;+\left\{0.07693^{*}\left[{\left(\frac{\text{BM}}{\text{height}}\right)}^{*}100\right]\right\} \end{array}$$

where BMO and GMO are, respectively, boys and girl's maturity offset; LL is leg length; SH is sitting height; A is age, and BM is body mass. With BMO and GMO data, any negative result is before PHV (maturity offset < 0, i.e., time left to reach the peak), and any positive results are after PHV (maturity offset = or > 0, i.e., indicating whether the participant is exactly at the beginning moment of PHV or how much this has passed). These equations are gender-specific, considering biological significance and statistics to predict maturity. Maturity offset indicates how far, in years, an age-group swimmer is approaching or moving away from PHV.

#### Statistical Analysis

Mean, SD and 95% confidence intervals were obtained and reported for all studied variables. Shapiro–Wilk test was applied to verify the data distribution, and comparisons were performed with paired-samples *t*-tests. In fact, gender as an independent variable was initially considered, but no significant effect was identified for any of the studied variables, possibly due to a similar maturation level of the participants. Therefore, we performed *t*-test comparisons instead of factorial ANOVA. Effect sizes were calculated from Hedges' g (Lakens, 2013) and interpreted with the following criteria: 0–0.19 trivial, 0.2–0.59 small, 0.6–1.19 moderate, 1.2–1.99 large, 2.0–3.99 very large, and ≥ 4.0 nearly perfect (Hopkins, 2002). Changes in % [Δ = (value after – value before)·100] were calculated for all variables.

To verify the associations among anthropometric, kinematics, and maturation variables changes, for both, BREAST and FLY, a machine learning technique (Network Analysis) was used (Epskamp et al., 2012). Gender was inserted in the network as a dichotomous variable (1 = girls and 2 = boys). In the network, variables were separated in Group 1, with gender and Δ of age, height, arm span, body mass; group 2 with Δ of T25, v, SR, SL, SI; and group 3 with just the Δ of the MO. Measures of centrality were generated to understand the role of each variable's change in the system, that is, the values are transformed into a z-score. We used three measures in our study (Epskamp et al., 2012):

(i) *Betweenness centrality*: estimated from the number of times that a node is part of the shortest path among all other pairs of nodes connected to the network.
(ii) *Closeness centrality*: determined from the inverse of the distances from one node to all others.
(iii) *Strength centrality:* the sum of all the weights of the paths that connect a node to the others.

We used the pairwise Markov random field model to improve the accuracy of the partial correlation network. The estimation algorithm used assumes the highest-order interaction of the true graph. The algorithm includes an L1 (regularized neighborhood regression) penalty. Regularization is achieved by a “less absolute contraction and selection operator” (LASSO) that controls the model's sparsity (Friedman et al., 2008). The Bayesian extended information criterion (EBIC) was used due to its conservative method for selecting the Lambda from the regularization parameter. The EBIC uses a hyperparameter (*y*) that determines how much the EBIC selects sparse models (Chen and Chen, 2008; Foygel and Drton, 2010). The *y* value is usually set between zero and 0.5; higher values indicate more parsimonious models with fewer edges, whereas a value closer to zero indicates an estimate with more edges. A *y* value of 0.25 is potentially useful for exploratory networks, and this value was adopted in our study (Foygel and Drton, 2010). The adjustment function returns the estimated parameters and a weighted and unweighted adjacency matrix. The positive relationships in the network are expressed in green and the negative in red. The thickness and intensity of the colors represent the magnitude of the associations. The “*graph*” package in the Rstudio software (http://www.rstudio.com/), and the “*qgraph*” package was used to construct the graphs (Epskamp et al., 2012).

## Results

Table 1 shows the results for anthropometrics and kinematics changes, effect sizes, and Δ. Small to moderate effect sizes were observed for changes on anthropometrical variables. Large effect sizes were observed for changes in nearly all kinematic variables, both in BREAST and in FLY. Performance of T25 in BREAST and FLY showed large improvements after 47 weeks. Only BREAST (trivial) and FLY (small) SL did not present at least moderate changes.

**Table 1:** BEFORE and AFTER 47 weeks (47w) mean ± SD values (95% confidence intervals), *p*-values, effects sizes (Hedges' g), and Δ% for anthropometric and performance/kinematics (*n* = 20).

	**Before 47 weeks**		**After 47 weeks**		***p*****-value; Effect size** Δ**% (before vs. after)**	
**Anthropometrics**						
Age (years)	10.2 ± 1.2 (9.6 to 10.8)		11.1 ± 1.2 (10.5 to 11.7)		<0.001; 0.75 (moderate) 8.9 ± 1.3	
Height (cm)	142.3 ± 9.7 (137.7 to 146.6)		147.8 ± 9.5 (143.1 to 151.9)		<0.001; 0.57 (small) 3.8 ± 1.4	
AS (cm)	143.6 ± 10.4 (138.5 to 148.1)		150.8 ± 11.3 (145.1 to 155.6)		<0.001; 0.41 (small) 4.9 ± 1.8	
BM (kg)	36.7 ± 8.2 (32.9 to 36.4)		41.4 ± 8.5 (37.0 to 45.0)		<0.001; 0.56 (small) 12.1 ± 6.7	
MO (years)	−2.0 ± 0.9 (−2.4 to −1.5)		−1.3 ± 1.0 (−1.8 to −0.8)		<0.001; 0.73 (moderate) 50.9 ± 281	
**Performance/** **kinematics**	**BREAST**	**FLY**	**BREAST**	**FLY**	**BREAST**	**FLY**
T25 (s)	32.0 ± 7.5 (28.4 to 35.7)	30.3 ± 7.0 (27.0 to 33.6)	24.5 ± 3.8 (22.7 to 26.4)	21.8 ± 3.6 (20.1 to 23.5)	<0.001; 1.26 (large) −21.9 ± 9.5	<0.001; 1.52 (large) −26.5 ± 11.0
*v* (m·s^−1^)	0.71 ± 0.12 (0.65 to 0.77)	0.76 ± 0.18 (0.68 to 0.85)	0.90 ± 0.12 (0.84 to 0.96)	1.05 ± 0.16 (0.97 to 1.12)	<0.001; 1.58 (large) 28.5 ± 19.7	<0.001; 1.70 (large) 41.0 ± 25.3
SR (cycles·min^−1^)	45.9 ± 11.9 (40.4 to 51.5)	37.1 ± 9.7 (32.5 to 41.6)	58.6 ± 8.6 (54.4 to 62.8)	45.6 ± 11.8 (40.0 to 51.1)	0.001; 1.22 (large) 26.7± 44.1	0.002; 0.78 (moderate) 16.3 ± 23
SL (m)	0.96 ± 0.23 (0.84 to 1.07)	1.30 ± 0.38 (1.13 to 1.48)	0.93 ± 0.13 (0.84 to 1.07)	1.43 ± 0.25 (1.31 to 1.55)	0.63; 0.16 (trivial) 2.2 ± 26.1	0.11; 0.40 (small) 16.7 ± 32.5
SI (m^2^·s^−1^)	0.69 ± 0.24 (0.58 to 0.81)	1.04 ± 0.41 (0.84 to 1.23)	0.84 ± 0.20 (0.75 to 0.94)	1.50 ± 0.35 (1.33 to 1.66)	0.005; 0.67 (moderate) 31.9 ± 49.2	0.001; 1.20 (large) 66.8 ± 77.4

*AS, arm span; BM, body mass; MO, maturity offset; T25, performance in 25-m; v, swimming speed; SR, stroke rate; SL, stroke length; SI, stroke index*.

**Figure 1:**
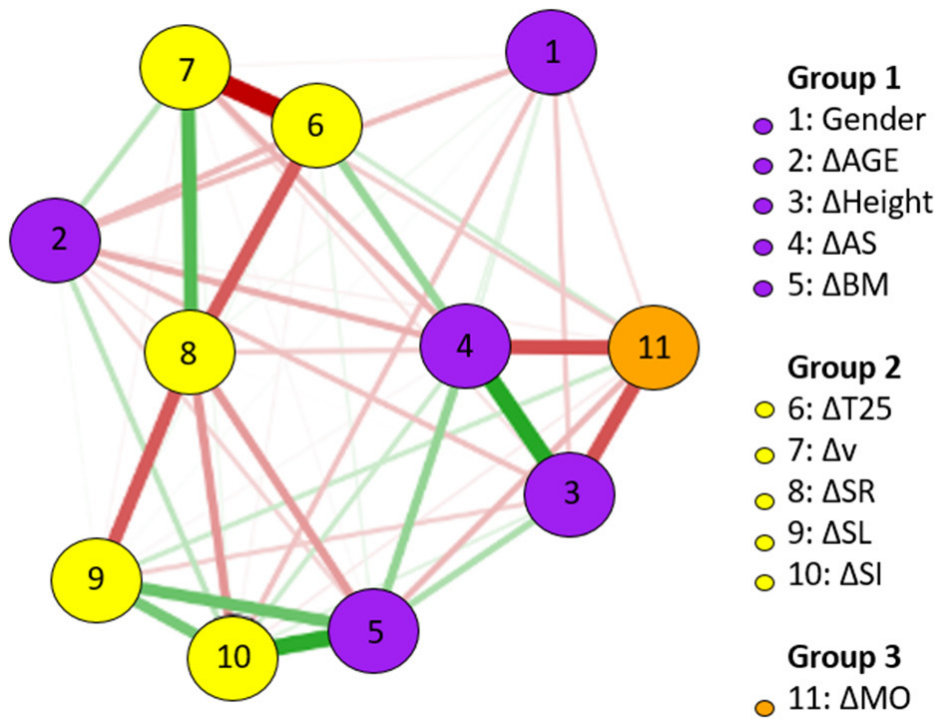
BREAST network of association between changes (Δ) in anthropometrics, maturation, and kinematics (using gender as a dichotomous variable); *n* = 20.

For BREAST, Figure 1 shows the network of association among changes in anthropometrics, maturation, and kinematics. Specifically in relation to the changes in T25 BREAST, the followings stand out: the strong and negative association with Δ*v*, the negative association with ΔSR, and the positive association with ΔAS. Positive and strong associations were identified between changes in height and AS, and between changes in AS and body mass. The relationship between changes in SL and SR was strong. Changes in SR showed more central associations inside the network.

The weight matrix for the BREAST is presented in Table 2. The results found for ΔMO and Δv (−0.93), ΔMO and ΔSR (−0.59), and for ΔSR and ΔSL (0.49) are highlighted.

**Table 2:** The weight matrix for the BREAST with the Δ% (gender as a dichotomous variable) (*n* = 20).

**BREAST**											
	**Gender**	Δ**Age**	Δ**Height**	Δ**AS**	Δ**BM**	Δ**MO**	Δ**T25**	Δ**v**	Δ**SR**	Δ**SL**	Δ**SI**
Gender	0										
ΔAge	−0.24	0									
ΔHeight	−0.19	−0.22	0								
ΔAS	0.10	−0.28	0.79	0							
ΔBM	0.09	−0.15	0.29	0.38	0						
ΔT25	0.01	−0.26	0.23	0.37	0.07	0					
Δ*v*	−0.03	0.24	−0.10	−0.27	−0.02	–**0.93**	0				
ΔSR	0.04	−0.08	−0.00	−0.07	−0.36	–**0.59**	0.63	0			
ΔSL	0.02	0.02	−0.17	−0.02	0.54	0.05	−0.03	–**0.60**	0		
ΔSI	−0.21	0.23	0.11	0.18	0.77	−0.04	0.05	−0.38	**0.49**	0	
ΔMO	−0.12	−0.05	−0.63	−0.64	−0.26	0.17	−0.18	−0.18	0.18	−0.07	0

*Δ, delta%; age; height; AS, arm span; BM, body mass; T25, performance in 25-m; v, swimming speed; SR, stroke rate; SL, stroke length; SI, stroke index; MO, maturity offset. Bold values that stand out in the analyzes*.

**Figure 2:**
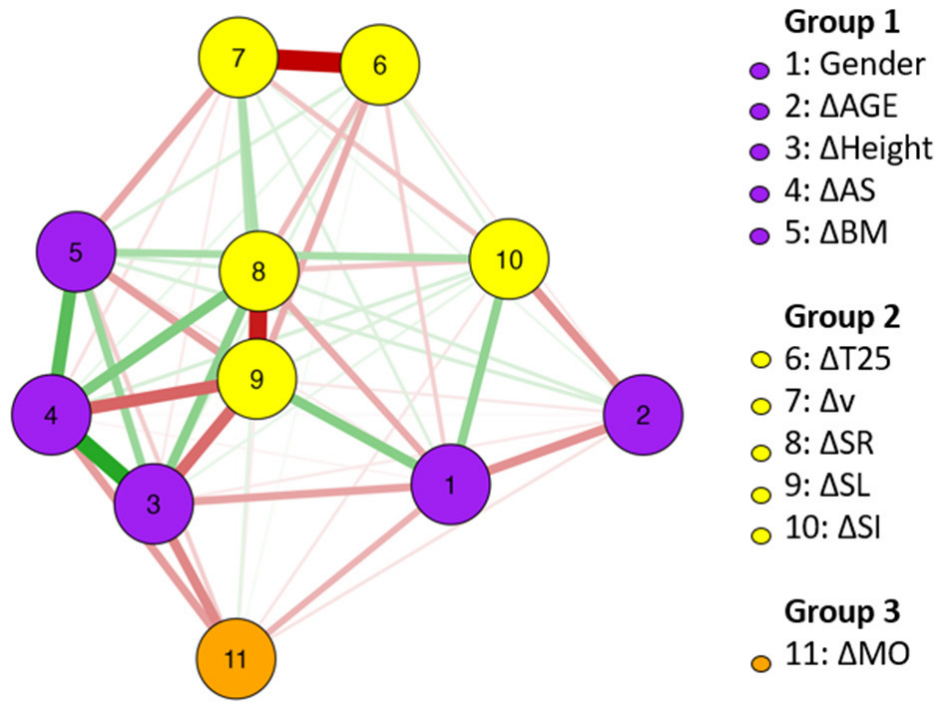
FLY network of association between changes (Δ) in anthropometrics, maturation, and kinematics (using gender as a dichotomous variable); *n* = 20.

For FLY, Figure 2 shows the network of association among changes in anthropometrics, maturation, and kinematics. Specifically in relation to the changes in T25 FLY, the followings stand out: the strong and negative association with Δ*v*, the negative association with ΔSR and ΔSL. Positive and strong associations were identified between changes in HE and AS, and between changes in AS and BM. The relationship between changes in SL and SR was strong. Changes in SR showed more central associations inside the network.

The weight matrix for the FLY is presented in Table 3. The results found for ΔMS and ΔSR (−0.92) are highlighted.

**Table 3:** The weight matrix for the FLY with the Δ% (gender (gender as a dichotomous variable) (*n* = 20).

**FLY**											
	**Gender**	Δ **Age**	Δ **Height**	Δ **AS**	Δ **BM**	Δ **T25**	Δ**v**	Δ **SR**	Δ **SL**	Δ **SI**	Δ **MO**
Gender	0.00										
Δ Age	−0.39	0.00									
Δ Height	−0.33	−0.08	0.00								
ΔAS	−0.04	−0.03	0.78	0.00							
ΔBM	−0.07	0.13	0.36	0.59	0.00						
ΔT25	−0.16	−0.07	0.10	0.08	0.13						
Δ*v*	0.11	0.07	−0.07	−0.11	−0.32	0.00					
ΔSR	−0.30	0.13	0.41	0.46	0.14	–**0.92**	0.00	0.00			
ΔSL	**0.43**	−0.08	–**0.53**	–**0.55**	−0.34	−0.24	0.30	−0.82	0.00		
ΔSI	0.39	−0.38	0.08	0.13	0.31	−0.30	0.27	−0.21	0.12	0.00	
ΔMO	−0.27	−0.10	−0.43	−0.35	−0.17	0.11	−0.21	0.10	−0.07	−0.09	0.00

*Δ, delta%; age; height; AS, arm span; BM, body mass; T25, performance in 25-m; v, swimming speed; SR, stroke rate; SL, stroke length; SI, stroke index; MO, maturity offset. Bold values that stand out in the analyzes*.

Table 4 shows the centrality measurements for BREAST and FLY. We highlight for betweenness, closeness, and strength ΔAS in BREAST and ΔSL in FLY. As gender is a dichotomous variable and does not suffer changes, those centrality measures will not be accounted.

**Table 4:** BREAST and FLY centrality measures (gender as dichotomous variable) (*n* = 20).

	**Betweenness**		**Closeness**		**Strength**	
	**BREAST**	**FLY**	**BREAST**	**FLY**	**BREAST**	**FLY**
Gender	−1.05	1.73	−2.39	0.31	−2.33	−0.01
ΔAge	−0.25	−0.83	−0.88	−1.44	−1.10	−1.56
ΔHeight	−0.25	0.02	−0.18	0.77	0.46	0.98
ΔAS	**1.34**	0.02	**1.19**	0.82	**1.11**	0.91
ΔBM	0.94	0.02	0.72	0.47	0.82	0.08
ΔT25	0.14	−0.83	0.67	−1.09	0.45	−0.53
Δ*v*	−1.05	−0.25	0.02	−0.74	0.08	−0.19
ΔSR	1.34	−0.83	0.30	0.71	0.82	0.89
ΔSL	−1.05	**2.02**	0.14	**1.67**	−0.52	**1.50**
ΔSI	0.94	−0.25	0.85	−0.49	0.14	−0.72
ΔMO	−1.05	−0.83	−0.46	−0.99	0.05	−1.34

*z-scored centrality metrics; Δ, delta%; age; height; AS, arm span; BM, body mass; T25, performance in 25-m; v, swimming speed; SR, stroke rate; SL, stroke length; SI, stroke index; MO, maturity offset. Bold values that stand out in the analyzes*.

## Discussion

We performed a global analysis to identify the relationships between changes in anthropometrics, maturation, and kinematics in 12-year-old and under age-group swimmers when swimming BREAST and FLY during a typical training season (47 weeks). The main finding of this study was that changes in performance and kinematics were higher than anthropometrics after 47 weeks, that is, improvements in swimming performance (T25) do not seem to be so dependent on growth, even though AS has stood out in the analysis of centrality measures.

Changes in technique (kinematics) may be related to motor coordination development in swimming (Guignard et al., 2017). Young swimmers are susceptible to change in their swimming mechanics at least three times in each competitive season (Morais et al., 2020). Typically, front crawl is the first swimming technique during swimming lessons in North America, whereas BREAST is the first in Europe, Asia, and Japan (Langerdorfer, 2013). However, Brazilian age-group swimmers composed our sample, where North America's learning sequence is normally followed. Thus, swimmers from our study were probably still in the process of learning simultaneous swimming techniques.

Simultaneous swimming techniques involve more coordinative skills and are less economic than alternate ones (Zamparo et al., 2020). BREAST is characterized by underwater recovery of both arms and legs (Leblanc et al., 2009), which produces resistive forces and consequently more energy expenditure (Zamparo et al., 2009). Other aspects that can influence poor glide and more exhausting action in BREAST are the head position combined with the breathing phase (Kapus et al., 2018) and the poor effectiveness of leg propulsion (Strzała et al., 2012). Similar events occur in FLY, in which both hands move to the surface from the water simultaneously (Thomas, 1990), something that tends to destabilize the positioning of the body (Sanders et al., 1995), turning the FLY into an “undulating stroke” (Riewald and Rodeo, 2015), characterized by the up- and downmovements of the body. Based on the motor coordination development, there are constraints in the motor learning process in the aquatic environment, which are visualized with the Newell (1986) model. Environmental, task, and organism factors may restrict the dynamic of the response, which could follow the reasoning about the process for improving simultaneous stroke performance.

The network analyses using changes in anthropometrics, maturation, and kinematics for both, BREAST and FLY, revealed the complexity of the systems. In swimming (Guignard et al., 2017), every action of a swimmer somehow disturbs the aquatic environment. This disturbance leads to new patterns of movement and so on. Likewise, a network analysis using data from a longitudinal approach that somehow can influence performance showed the multiple associations between changes after 47 weeks on anthropometric and kinematic variables. Even that, changes in T25 were mainly linked to Δ*v*, ΔSR, and ΔAS for BREAST, and Δ*v*, ΔSR, and ΔSL for FLY. The notion of complexity on changes in swimming performance was reinforced, especially for simultaneous techniques in age-group swimmers.

Regarding centrality, the betweenness indicates which variables are closer to others and could be the easiest path for changes. In the BREAST, changes in AS and SR (both 1.34) and SI (0.94) were highlighted. Clearly, AS changes are beyond intervention possibilities. However, the focus on SR and stroke index (SI, an indirect measure swimming efficiency) to a given v (Costill et al., 1985) seems to be important in BREAST performance development. Regarding FLY, changes in the SL (2.02) are probably related to the variation in the distance covered per cycle, but in a specific way; that is, the complex coordination between one arm stroke, one undulation, and two kicks could be executed in an easier way by young swimmers (Tosta et al., 2019), which implies an improved performance.

The closeness measure can indicate which variables could be more quickly affected by interventions. Regarding BREAST, changes in AS and SR (both 1.34) and SI (0.94) were highlighted. Since AS is an anthropometric variable, the focus for faster changes in performance in BREAST should be on changes in SR and SI (a variable that incorporates both SL and v) (Costill et al., 1985). Regarding FLY, as in the betweenness measure, changes in SL are dominant in performance changes. The strength measure indicates which variables (in the current pattern of the network) have the strongest relationships. For both BREAST and FLY, changes in AS showed high values of strength. However, changes in SR (for BREAST) and SL (for FLY) were also highlighted. All these centrality measures must be analyzed under the environment constraint theory (Newell, 1986).

According to Newell (1986), environment constraints refer to the environmental conditions surrounding the subject and can be physical or social, such as the aquatic milieu, water and air temperature, and audience, among others. Establishing oneself as an independent individual in the aquatic milieu is a long and necessary process to become a swimmer. This skill mastery requires repetitive exercise for a certain time before actually mastering it (Gani et al., 2019). The individual thus acquires “water sensitivity” and can properly use his or her body dimensions and propulsive force to advance. This relationship was evident for FLY, mainly due to changes in SL. Perhaps, changes in SL were related to the undulation, that is, the FLY leg kick. This movement is not a “natural” movement for humans, that is, it involves an individual adaptation with the environment combined with a development in motor skills. Over time, children replace the “pedaling” movement of the legs by oscillation of the flippered foot (Collard et al., 2013), which leads to the issue of task constraints.

Task constraints describe the activity to be performed by the subject and whether individual objectives, rules or instructions, and possible implements are included. Task constraints can generate changes in movement patterns, and these changes trigger changes in the system, which leads individuals to a new organizational state (Newell, 1986). Synchronization between specific motor points of arm and leg actions are the key factor for fast FLY swimming (Strzała et al., 2017). Technical development provides a more economic technique, using less force for a determinant movement. Previous data by Havriluk (2010) indicated that the advantage of faster swimmers derives more from technique than force capacity.

Typically, beginner swimmers spend more time with the head out of water during breathing time when swimming BREAST. It has been well reported that head position influences technique (Kapus et al., 2018), and leg glide is significantly smaller among nonexperienced swimmers (Leblanc et al., 2009). The authors observed that recreational swimmers perform BREAST arm recovery while doing their leg kick, which shows a simultaneous extension of their two pairs of limbs. In addition, novice swimmers are prone to not pull with their arms while recovering their legs (Taguchi, 1975). These actions are related to changes in motor skills which are developed during training sessions (Table 1). Moreover, impaired SL combined with increased SR when comparing before and after may be related to less time spent during breathing time when swimmers are more experienced, making the stroke more cyclic and adjusted to the T25 pace.

Organism constraints refer to the characteristics of the subject (Newell, 1986), such as anthropometric, physiological, and psychological factors. Changes in AS for both BREAST and FLY presented a high strength value (Table 4) and was one of the variables with the strongest associations inside the network. A previous study (Sammoud et al., 2018) indicated that fat mass is the most important whole-body size characteristic for 100-m BREAST (~12 years old) and was one of the variables with the strongest associations inside the network. Sammoud et al. (2017) suggested that anthropometric measurements are strongly associated with the 100-m butterfly speed performance of age-group swimmers (~13 years old). High-level swimmers present a wider AS, imposing higher *v* and SI, and therefore, faster performance (Sammoud et al., 2017) than those with shorter AS. These findings highlight that anthropometric factors are somehow related to the performance changes during a training season in age-group swimmers.

Maturity offset can play a role in the organism constraints for stroke coordination. For BREAST and FLY, changes in MO showed a high relationship with the development process, with high values for betweenness (Table 4), that is, puberty affects swimming technique development. Likewise, in FLY, changes in MO showed higher values of closeness and strength. Swimmers with a more advanced maturation status presented better coordination when swimming FLY than others (Tosta et al., 2019). However, although maturation of prepubertal swimmers seems to be an important factor for consideration in FLY stroke coordination, it does not affect the maximum performance for short distances (Tosta et al., 2019). Despite some correlation between changes in AS and changes performance of T25, kinematics (SR and SI) better explained swimming performance.

The swimming athletic development process is multifactorial (Zacca et al., 2020a). Coaches should be aware of their athletes' maturation processes, understand the impact of growth on changes in performance, and seek the best swimming technique (Zacca et al., 2020b). However, looking at all factors, as a global model, is fundamental to understanding swimmer's development (Goethel et al., 2020). Knowing how to handle changes in SR and SL and considering body growth and maturation can help in LTAD strategies for swimming and related aquatic sports.

The use of network analysis to understand a phenomenon in sports and health sciences is quite new, but its basic ideas have been noted since the 1960s (Grusky, 1963). The digital technology development has contributed to an exponential increase in network analysis studies in health and sport sciences (Wäsche et al., 2017; Goethel et al., 2020; Lord et al., 2020). Although network analysis offers advantages compared to traditional statistical procedures, it is important to acknowledge some shortcomings and potential limitations. Network analysis, a set of integrated techniques, was applied in this study trying to describe relations among variables, by analyzing the structures that emerge from the recurrence of these relations. When performing a network analysis, it is assumed that better interpretations of phenomena are yielded. Despite that, causal relationships between networks and a specific phenomenon normally involve a theoretically informed decision that identifies the independent and dependent variables. Whereas deterministic methods usually highlight that network analysis enables to study how the structure of relationships affectsthe phenomena, “structurally bounded purposive actions may affect the social structure and vice versa (Chiesi, 2001). The sample size can be a problem for estimating networks with many parameters and consequently for interpretation. To increase reliability and limit the number of possibly spurious relationships in the network, we use statistical regularization techniques that consider the complexity of the model to minimize the small sample. First, we used a LASSO (Friedman et al., 2008) applied to the estimation of partial correlation networks. LASSO performs well in estimating partial correlation networks (Fan et al., 2009), and this results in some small weak edge estimates being reduced to exactly zero, resulting in a sparse network (Tibshirani, 1996). LASSO generates a tighter graph (fewer connections between nodes), reflecting merely the most important empirical relationships in the data. Simulation studies suggest that LASSO has a low probability of false positives, which provides some confidence that an observed edge is indeed present in the network in small samples (Krämer et al., 2009). Besides, LASSO requires the definition of a tuning parameter. The sparsity of the produced network by LASSO depends on the value that the researcher sets the fitting parameter (λ), that is, the higher the selected λ value, the more edges are removed from the network, Thus, its value directly influences the structure of the output (i.e., the network). Thus, the fitting parameter “λ” needs to be carefully selected to generate a network structure that minimizes the number of spurious edges while maximizing the number of true edges (Foygel and Drton, 2010). To ensure an optimal fitting selected parameter, a typical method includes estimating multiple networks under different λ values. These different networks range from a completely connected network (where each node is connected to each other) to an empty network (where no nodes are connected). LASSO estimations generate a collection of networks rather than a single network, that is, it is important to select the ideal network model, which is usually achieved by minimizing the “extended Bayesian information criterion” (EBIC) (Chen and Chen, 2008), which works well in identifying the true network structure (Foygel and Drton, 2010; van Borkulo et al., 2014), especially when the true network is scarce. EBIC has been extensively used in psychology networks (e.g., Beard et al., 2016; Isvoranu et al., 2016), preschoolers (Bandeira et al., 2020; Martins et al., 2021) by increasing the accuracy and interpretability of generated networks (Tibshirani, 1996). Thus, although network models can be reliable and robust with small samples, this aspect may be a limitation in our study. Finally, studies with older swimmers and/or adding physiological related variables (e.g., metabolic power and energy cost; di Prampero, 1986; Zamparo et al., 2020; Zacca et al., 2020b) will be welcomed in the next related experiments.

Twelve-year-old and under age-group swimmers regularly change their technique when swimming BREAST and FLY. Maturation, HE, AS, and SL showed a great impact on BREAST development, whereas age, SR, and HE had a strong impact for FLY. The SI represents an indirect measure of swimming efficiency and should be monitored in both BREAST and FLY to connect growth with the other technique variables. The dynamic process of athletic development and the perception of complexity of changes and relationships between swimming performance-related variables were underpinned, particularly for simultaneous techniques in age-group swimmers.

## Data Availability Statement

The raw data supporting the conclusions of this article will be made available by the authors, without undue reservation.

## Ethics Statement

The studies involving human participants were reviewed and approved by Universidade Federal do Rio Grande do Sul. Written informed consent to participate in this study was provided by the participants' legal guardian/next of kin.

## Author Contributions

JF, PB, RZ, and FC developed the original research inquiry and analyzed data, collaborated in data interpretation, writing, and reviewing the manuscript. JF and FC recruited participants and collected data. All authors approved the final version of this manuscript.

## Funding

This publication was funded by Universidade Federal do Rio Grande do Sul, Brazil. RZ is funded by Research Center in Physical Activity, Health and Leisure—CIAFEL - Faculty of Sports, University of Porto—FADEUP (FCT UID/DTP/00617/2020 and Laboratory for Integrative and Translational Research in Population Health (ITR), Porto, Portugal (LA/P/0064/2020).

## Conflict of Interest

The authors declare that the research was conducted in the absence of any commercial or financial relationships that could be construed as a potential conflict of interest.

## Publisher's Note

All claims expressed in this article are solely those of the authors and do not necessarily represent those of their affiliated organizations, or those of the publisher, the editors and the reviewers. Any product that may be evaluated in this article, or claim that may be made by its manufacturer, is not guaranteed or endorsed by the publisher.
